# Adjuvant Mucosal Strategies Confer Safe and Effective Immunity Against *Mycoplasma pneumoniae* and Overcome Vaccine-Associated Enhanced Lung Pathology

**DOI:** 10.3390/vaccines13090968

**Published:** 2025-09-12

**Authors:** Zhentao Lei, Dandan Gao, Xiaolong Zhang, Han Cao, Jingping Hu, Yifan Zhou, Ning Luan, Cunbao Liu

**Affiliations:** Institute of Medical Biology, Chinese Academy of Medical Sciences and Peking Union Medical College, Kunming 650118, China; s2023018021@pumc.edu.cn (Z.L.); ddgao2008@imbcams.com.cn (D.G.); zhangxiaolong@imbcams.com.cn (X.Z.); caohan@imbcams.com.cn (H.C.); hujingping@student.pumc.edu.cn (J.H.); mickey6zyf@163.com (Y.Z.)

**Keywords:** *Mycoplasma pneumoniae* (MP), inactivated vaccine, enhanced lung pathology, FLA-ST, mucosal immune response

## Abstract

Background/Objectives: The global spread of *Mycoplasma pneumoniae* (MP) poses a significant threat to public health; however, no licensed vaccine for human use is currently available. The development of a safe and effective vaccine is a critical priority. This study systematically evaluated the protective efficacy and safety of an inactivated MP vaccine using different adjuvants and immunization routes. Methods: Mice were immunized with inactivated vaccines via either intramuscular (IM) injection with aluminum hydroxide (alum) or a combination of CpG+QS21 (CQ) or via intranasal (IN) administration of Flagellin from *Salmonella Typhimurium* (FLA-ST), a potent Toll-like receptor 5 (TLR5) agonist, as a mucosal adjuvant. Vaccine-induced immunogenicity, protective efficacy against MP challenge, and associated lung pathology were assessed. Results: Both IM-vaccinated groups (alum and CQ) exhibited robust systemic immune responses. However, upon subsequent MP challenge, these groups exhibited significant inflammatory pathology in the lung tissues. Notably, the CQ-adjuvanted group displayed severe pulmonary inflammatory infiltration. In stark contrast, compared with the IM-vaccinated group, the IN-immunized group with the FLA-ST mucosal adjuvant achieved significant clearance of MP from the lungs and showed markedly milder histopathological lung damage. Conclusions: Our findings suggest that IM immunization with CQ-adjuvanted inactivated vaccines may represent a suboptimal strategy for MP, given the risk of exacerbating lung immunopathology. Conversely, a mucosal immunization approach using the FLA-ST adjuvant demonstrates considerable promise, offering an effective balance between bacterial clearance and an improved safety profile, highlighting its potential for future MP vaccine development.

## 1. Introduction

*Mycoplasma pneumoniae* (MP) is a unique prokaryote characterized by the absence of a cell wall and is the smallest known pathogen capable of growth and replication in cell-free media [1]. Infection is transmitted primarily via respiratory droplets, with coughing and sneezing as the main modes of transmission. MP is prevalent globally throughout the year, with peak incidence observed during the autumn and winter [2,3]. As a major causative agent of community-acquired pneumonia (CAP), MP can infect individuals of all age groups, accounting for up to 40% of CAP cases in children and adolescents [4,5].

Infection with MP can result in a wide spectrum of clinical presentations, ranging from mild upper respiratory tract infection to MP pneumonia (MPP). Furthermore, it can lead to a range of extrapulmonary manifestations, such as rash and mucositis, myocarditis, erythema multiforme, and meningoencephalitis, through hematogenous dissemination or an excessive immune response [6,7,8,9]. MP pathogenesis is intricately linked to its unique cellular structure. Key adhesion proteins, such as P1 and P30, located at its terminal organelle, mediate the tight adherence of the pathogen to host respiratory epithelial cells, subsequently causing cellular damage and an inflammatory response [10,11,12].

Currently, macrolide antibiotics, such as erythromycin and azithromycin, constitute the mainstays of clinical treatment for MPP [13]. However, the extensive use of these antibiotics has led to a progressive increase in macrolide resistance in MP, with rates now exceeding 90% in some parts of Asia [14,15]. This increase in drug resistance poses a significant challenge to the clinical management of MPP and highlights the urgent need for the development of an effective vaccine for the prevention of MP infection.

Since the 1960s, initial efforts to develop a vaccine against MP have centered on traditional platforms, including inactivated and live attenuated vaccines [16,17]. Nevertheless, these early endeavors encountered significant setbacks. The risk of vaccine-associated enhanced respiratory disease (VAERD) represents a formidable and long-standing challenge in the development of effective MP vaccines. This concern is rooted in seminal early research, such as the 1967 human volunteer study by Smith et al., which first documented that vaccinated individuals who failed to mount a significant serological response subsequently experienced more severe illness upon experimental challenge than their unvaccinated counterparts did [16]. This historical observation is acknowledged and reinforced by contemporary studies, which cite this early work and further demonstrate in animal models that an inactivated vaccine, while reducing bacterial load, can paradoxically fail to suppress detrimental pulmonary inflammation, such as neutrophil infiltration [18]. More recent mechanistic research has begun to elucidate the “women” behind this phenomenon, suggesting that VAERD is driven by a skewed Th17 immune response, likely induced by vaccine components such as lipoproteins. The aberrant response primes the host for excessive, IL-17A-driven neutrophilia upon subsequent infection, which mediates the severe immunopathological characteristics of VAERD instead of conferring protection. Collectively, these studies consistently highlight the risk that inactivated MP vaccines may induce harmful immune priming that exacerbates disease [19]. Concurrently, live attenuated vaccine candidates have not advanced to clinical use owing to safety concerns regarding their potential for reversion to a virulent phenotype [20]. More recently, the focus of vaccine development has pivoted toward next-generation platforms, such as subunit and nucleic acid-based vaccines, which offer improved safety and precision [21,22]. However, these approaches, although promising in principle, are constrained by formidable challenges, including prohibitive costs, complex manufacturing, and, crucially, suboptimal immunogenicity. As a result, currently, a licensed MP vaccine is not available globally [20,23]. This state of affairs underscores the inadequacies of current paradigms and necessitates an urgent search for more innovative and effective solutions.

To address the challenge of insufficient immunogenicity and induce more potent and durable immune protection, this study employed a strategy that combines a novel adjuvant system and an optimized immunization route with an inactivated MP vaccine. First, instead of the traditional aluminum adjuvant, we incorporated a composite adjuvant consisting of CpG oligodeoxynucleotides (CpG ODN, briefly called CpG below) and QS-21, a mixture of saponins extracted from the bark of *Q. saponaria*. As an agonist of toll-like receptor 9 (TLR9) [24], CpG induces a strong Th1-type immune response by activating the downstream MyD88 signaling pathway, which is characterized by the production of high levels of IFN-γ and IL-12 [25,26]. QS-21 is a potent immunostimulant that enhances antigen-specific antibody responses and effectively induces the generation of cytotoxic T lymphocytes (CTLs), thereby inducing a robust cellular immune response [27,28]. The synergistic action of CpG and QS-21 (CQ), which activates multiple innate immune pathways to elicit high-titer antibodies and a robust CTL-mediated cellular response, has been successfully applied in the shingles vaccine [29]. However, its application in the context of inactivated MP vaccines remains unexplored. Second, given that MP is a respiratory pathogen, inducing local mucosal immunity is crucial for preventing infection [30,31]. Therefore, this study further explored intranasal (IN) immunization, utilizing bacterial flagellin (FLA-ST) as a mucosal adjuvant to assess its ability to induce local protective immunity. FLA-ST, a classic pathogen-associated molecular pattern (PAMP), acts as an adjuvant by binding to toll-like receptor 5 (TLR5) on the cell surface, which initiates downstream signaling cascades to effectively activate both humoral and cellular immunity [32,33,34].

## 2. Materials and Methods

### 2.1. Animals and Ethical Statement

A total of 30 specific-pathogen-free (SPF) female BALB/c mice (4–6 weeks old) were purchased from Sipeifu (Beijing, China) Biotechnology Co., Ltd. (License No. SCXK (Chuan) 2023-0040). The mice were randomly divided into six groups (n = 5 per group), comprising four experimental groups, one challenge control group, and one negative control group. All mice were housed in the Animal Experiment Center of the Institute of Medical Biology, Chinese Academy of Medical Sciences (IMB, CAMS). The facility provided SPF conditions, including a circulating ventilation system, controlled temperature, and humidity. The animals had ad libitum access to sterile food and water. Following a one-week acclimatization period, the experiments commenced. All animal procedures were reviewed and approved by the Animal Welfare and Ethics Committee of the IMB, CAMS (Ethics and Biosafety Approval No. DWSP202405005/SWAQ20240802).

### 2.2. Bacterial Strain and Culture Conditions

The MP M129 standard strain (ATCC 29342) used in this study was maintained in our laboratory. The basal medium was prepared using PPLO Broth Base (BD, Franklin Lakes, NJ, USA) and yeast extract powder (Oxoid, Basingstoke, Hampshire, UK), with phenol red (Coolaber Science & Technology, Beijing, China) as a pH indicator, and sterilized by autoclaving. After the medium cooled to 40–50 °C, it was aseptically supplemented with 20% (*v*/*v*) sterile fetal bovine serum (FBS; VIVACELL; Biochrom GmbH, Berlin, Germany) and 5% (*w*/*v*) sterile dextrose solution (Tianjin Fengchuan Chemical Reagent, Tianjin, China). The final pH of the complete medium was adjusted to approximately 7.9. MP stock was inoculated into complete PPLO medium at a 1:10 (*v*/*v*) ratio and cultured at 37 °C in a humidified 5% CO_2_ atmosphere using a CO_2_ incubator (Model: CLM-170B-8-CN; Thermo Fisher Scientific, Waltham, MA, USA). Bacterial growth was monitored daily by observing the color of the medium; a shift from red to orange or yellow indicated robust MP proliferation due to acid production. To harvest the bacteria, the culture was centrifuged at 12,000 rpm for 20 min at 4 °C. The supernatant was discarded, and the bacterial pellet was resuspended in sterile phosphate-buffered saline (PBS). For quantification, a 50 µL aliquot of the suspension was spread onto PPLO solid agar plates and incubated at 37 °C for 5–7 days in a microaerophilic atmosphere (5% CO_2_, 95% N_2_). The resulting colonies were counted under an inverted microscope (Model: DMi1; Leica Microsystems, Wetzlar, Germany) to determine the concentration in colony-forming units (CFUs). The final bacterial stock was adjusted with sterile PBS to a concentration of 10–10^9^ CFU/mL and stored in aliquots at −80 °C until use.

### 2.3. Preparation of Inactivated MP Vaccines

MP was initially subcultured in small volumes (5–10 mL) according to the culture methods and conditions described previously. When the color of the liquid medium turned orange–yellow, indicating the logarithmic growth phase, the culture was passaged at a 1:10 ratio and scaled up. The process was continued until a final volume of 1 L of Mp culture in the logarithmic phase (characterized by an orange-yellow medium) was harvested. For inactivation, 1 mL of β-propiolactone (J&K Scientific Ltd., Beijing, China) was added to the 1 L culture to a final concentration of 0.1% (*v*/*v*). The mixture was incubated at room temperature for 10 h to ensure complete inactivation. The inactivated Mp culture was then centrifuged at 12,000 rpm for 30 min at 4 °C. The resulting bacterial pellet was subsequently washed twice and resuspended in sterile PBS. The protein concentration of the inactivated MP antigen was quantified using a bicinchoninic acid (BCA) protein assay (Beyotime, Beijing, China). The final concentration was determined to be 1.27 mg/mL.

### 2.4. Immunization and Challenge

The experimental groups received an inactivated vaccine formulated as follows: (1) the adjuvanted-free group received an intramuscular (IM) injection of inactivated MP vaccine (20 µg MP antigen/total volume of 100 μL/mouse); (2) the alum group received an IM injection of inactivated MP vaccine adjuvanted with alum (ThermoFisher Scientific, Cat# 77161, now discontinued) (20 µg MP antigen/50 µg alum/total volume of 100 μL/mouse); (3) the CQ group received an IM injection of inactivated Mp vaccine adjuvanted with a combination of 10 μg/dose CpG ODN 1018 (5′-TGACTGTGAACGTTCGAGATGA-3′; synthesized by Sangon Biotech, Shanghai, China) and 5 μg/dose QS21. Alpha Diagnostic International, Inc., San Antonio, TX, USA); (4) the FLA-ST group received the inactivated MP vaccine co-administered with FLA-ST adjuvant (5 µg/mouse, InvivoGen, San Diego, CA, USA) via intranasal (IN) instillation in a 100 µL dose; (5) the PBS group and (6) the blank control group received sterile PBS at the same volume.

A booster immunization was administered four weeks after the primary immunization using the same route and dosage for each respective group. Two weeks after the final immunization, blood samples were collected, and the immunized serum was harvested. The mice in the first five groups were subsequently challenged by IN instillation of 50 µL of a live MP suspension (1 × 10^9^ CFU/mL), whereas the blank control group received the same volume of PBS. Following the challenge, the mice were monitored daily for changes in body weight, fur condition, clinical signs of disease, and overall survival rate.

### 2.5. Measurement of Specific Antibody Titers

Serum levels of MP-specific IgG, IgG1, and IgG2a were determined using enzyme-linked immunosorbent assays (ELISAs). Additionally, secreted IgA (sIgA) levels were measured in eluate from throat swabs and vaginal swabs and in bronchoalveolar lavage fluid (BALF) collected 7 days postchallenge. For the ELISA, 96-well plates were coated with 50 µL/well of whole-cell MP protein antigen (6 µg/mL) and incubated overnight at 4 °C. The antigen coating was prepared by sonicating the MP suspension on ice for a total duration of 2 h, with 20 s of sonication followed by 40 s of rest. The next day, the plates were washed with PBST (PBS with 0.05% Tween-20) and blocked with 150 µL/well blocking buffer (5% nonfat milk in PBS) for 1 h at 37 °C. Serum and BALF samples were serially diluted with dilution buffer (1% nonfat milk in PBS), starting from a 1:10 dilution, whereas samples from throat swabs and vaginal swabs were diluted twofold in PBS containing 1% nonfat milk, starting from a 1:2 dilution. The samples were added to the wells and incubated for 1 h at 37 °C. After the cells were washed, 50 µL/well of HRP-conjugated secondary antibodies (goat anti-mouse IgG, IgG1, IgG2a, or IgA; Invitrogen, Hercules, CA, USA) were added, and the samples were incubated for 1 h at 37 °C. The plates were subsequently washed again, and 50 µL/well of TMB substrate solution (Solarbio Science & Technology Co., Ltd., Beijing, China) was added for color development in the dark for 10 min. The reaction was terminated by the addition of 50 µL of 2 M H_2_SO_4_. The optical density (OD) at 450 nm was measured using a microplate reader. End-point titers were defined as the highest serum dilution yielding an OD value ≥ 2.1 times that of the PBS control.

### 2.6. Quantification of the MP Bacterial Load in Lung Tissue

To determine the bacterial burden in the lungs, the mice were euthanized, and the lung tissues were aseptically harvested and weighed. Each lung was placed in a sterile grinding tube containing 1 mL of sterile PBS and steel beads. The tissues were homogenized using a tissue lyser (Model: JXFSTPRP; Shanghai Jingxin Industrial Development Co., Ltd., Shanghai, China) at a frequency of 60 Hz for 30 s. The resulting lung homogenates were centrifuged at 3000 rpm for 10 min at 4 °C to pellet tissue debris. A 50 µL aliquot of the supernatant from each sample was then evenly plated onto PPLO solid agar plates. The plates were incubated at 37 °C for 5–7 days. Following incubation, the characteristic “fried-egg” MP colonies were observed and counted under an inverted microscope at low magnification. The bacterial load was calculated and expressed as CFU/g lung tissue.

### 2.7. Determination of MP Load in BALF by qPCR

The BALF samples were centrifuged at 2000 rpm for 10 min at 4 °C to separate the supernatant from the cellular components. Genomic DNA was subsequently extracted from the supernatant using a Blood/Cell/Tissue Genomic DNA Extraction Kit (Servicebio, Wuhan, China) according to the manufacturer’s instructions. The quantity of MP genomic DNA in the BALF was determined by quantitative real-time PCR (qPCR). The specific primers and probe used for the amplification of the MP gene were as follows: forward primer, 5′-CCAACCAAACAACAACGTTCA-3′; reverse primer, 5′-ACCTTGACTGGAGGCCGTTA-3′; and probe, 5′-FAM-TCAATCCGAATAACGGTGACTTCTTACCACTG-BHQ1-3′.

### 2.8. Histopathological Analysis of Lung Tissue

Following euthanasia, the lungs were aseptically excised from the mice and immediately fixed in a 4% paraformaldehyde solution (Biosharp, Hefei, China). The fixed tissues were then sent to Servicebio (Wuhan, China) for standard histological processing, including paraffin embedding, sectioning, and hematoxylin and eosin (H&E) staining. The stained sections were examined for histopathological changes. A pathologist, who was blinded to the experimental groups, evaluated and scored each slide to quantify the degree of lung injury. The scoring was based on several key pathological features, including inflammatory cell infiltration in the alveolar and bronchial regions, interstitial congestion and hemorrhage, thickening of the alveolar wall, and connective tissue proliferation or fibrosis. A 5-point grading scale (0–4) was used as follows:

Score 0: Normal; no pathological changes were observed.

Score 1: Very mild; lesions are minimal and just beyond the normal range.

Score 2: Mild; lesions are clearly observable but not severe.

Score 3: Moderate; lesions are distinct, prominent, and widespread.

Score 4: Severe; lesions are very severe and affect a large portion of the lung tissue.

### 2.9. Data Analysis

Statistical analyses were performed using GraphPad Prism version 10.1.2 (GraphPad Software, La Jolla, CA, USA). Significant differences among groups were assessed by one-way analysis of variance (ANOVA), which was followed by Tukey’s multiple comparisons test to determine the significance of differences between the means of individual groups. Significance is denoted as follows: ns for *p* > 0.05; * for *p* < 0.05; ** for *p* < 0.01; *** for *p* < 0.001; and **** for *p* < 0.0001.

## 3. Results

### 3.1. Compared with Other Adjuvanted MP Vaccines, FLA-ST Resulted in Lower Pathogen-Specific Antibody Titers

**Figure 1:**
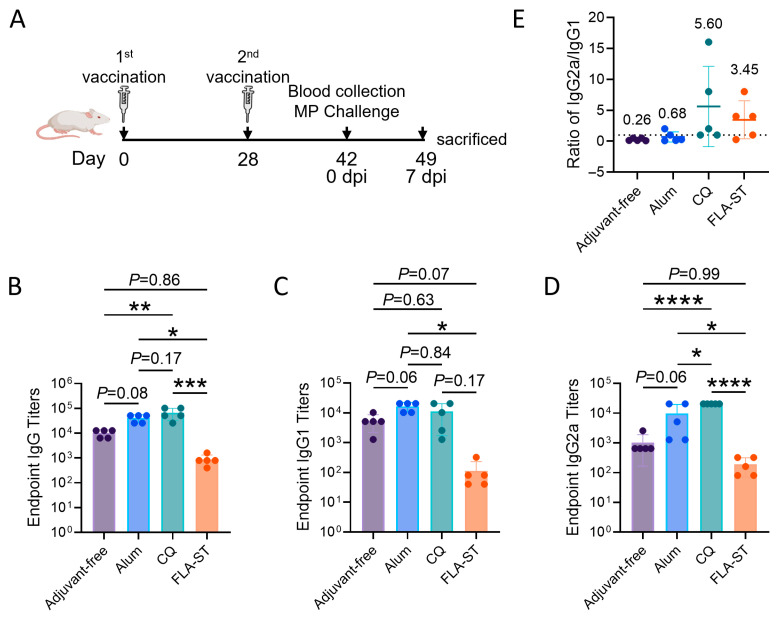
Humoral immune responses induced by the candidate vaccine formulated with different adjuvants: (**A**) Schematic diagram of the mouse immunization and MP challenge procedure. (**B**–**D**) Antigen-specific total IgG (**B**), IgG1 (**C**), and IgG2a (**D**) antibody titers in the serum of immunized mice were determined by ELISA at the end time point (Day 42). (**E**) The ratio of IgG2a/IgG1 titers was calculated to evaluate the Th1/Th2 polarization of the immune response. The dashed line represents the ratio of 1 (IgG1 = IgG2a), and the mean values were added for clear illustration. The data are shown as the mean ± SD. Significant differences between the four vaccinated groups were assessed by ANOVA, and the mean of each column was compared with the mean of every other column. *, *p* < 0.05; **, *p* < 0.01; ***, *p* < 0.001; ****, *p* < 0.0001. MP, *Mycoplasma pneumoniae*.

To characterize the systemic humoral immune profiles, the mice were immunized twice, with serum collected two weeks after the final immunization for antibody analysis, followed by pathogen challenge and endpoint analysis on Day 7 post infection (experimental schematic, Figure 1A). First, we assessed the magnitude of the serum antibody response. As anticipated, IM immunization with the CQ adjuvant robustly induced the highest titers of total IgG, IgG1, and IgG2a. Conversely, compared with all the other immunized groups, intranasal immunization with the FLA-ST adjuvant resulted in significantly lower levels of these systemic antibodies (Figure 1B–D). T-helper (Th) polarization of the immune response was investigated by calculating the IgG2a/IgG1 ratio (Figure 1E). Consistent with previous reports, the CQ adjuvant promoted a strong Th1-biased response, with a mean IgG2a/IgG1 ratio of 5.6. Notably, the mucosal FLA-ST group also induced a response predominantly skewed toward a Th1 phenotype, with a mean IgG2a/IgG1 ratio of 3.45. In stark contrast, both the alum-adjuvanted and adjuvant-free IM groups, which induced antibody levels comparable to each other, elicited clear polarization toward a Th2-type response, with an IgG2a/IgG1 ratio lower than 1. This distinction is critically important, as prior research has linked Th17-skewed immunity with an increased risk of VAERD following MP challenge [19].

### 3.2. The FLA-ST Adjuvant Combined with Mucosal Immunization Provides Both Safe and Efficacious Protection

Following the immunization schedule, we evaluated the in vivo protective efficacy of each vaccine formulation by challenging mice with MP. Clinical signs, survival, and pulmonary bacterial burden were monitored to assess outcomes.

**Figure 2:**
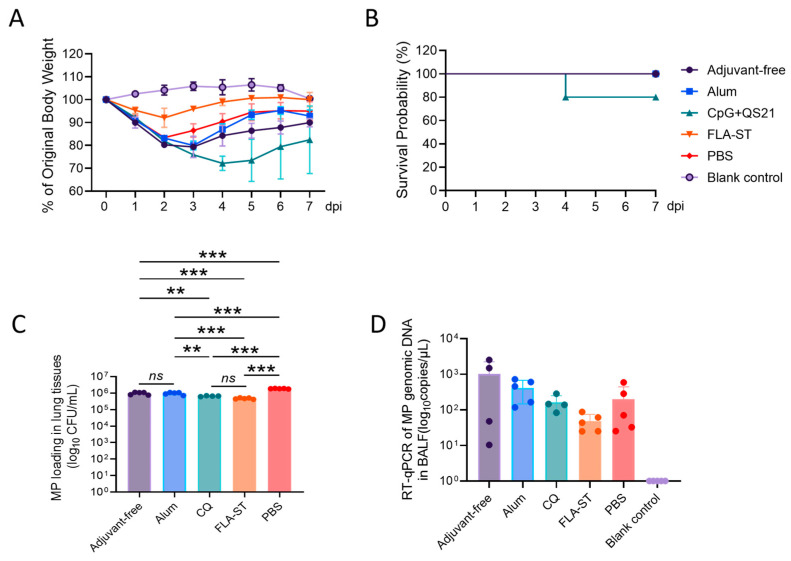
Body weight changes and survival rates of mice after MP challenge and pathogen loading in the lung tissues of mice after challenge: (**A**) Percentage change in body weight relative to the initial weight. (**B**) Survival rates of mice after MP challenge. (**C**) MP colony counts in the lung tissues of mice 7 dpi. (**D**) MP loading in the BALF at 7 dpi, as determined using qPCR. Significant differences between the four vaccinated groups were assessed using ANOVA, and the mean of each column was compared with the mean of every other column. ns, not significant; **, *p* < 0.01; ***, *p* < 0.001. MP, *Mycoplasma pneumoniae*; BALF, bronchoalveolar lavage fluid.

First, we assessed morbidity and mortality by tracking daily body weight and survival for seven days. The PBS group exhibited severe morbidity, with body weight reaching a nadir of 83.4% of the initial value at 2 dpi (Figure 2A). The alum-adjuvanted and adjuvant-free groups showed similar patterns of weight loss and recovery, which is consistent with their comparable systemic IgG responses (Figure 1B and Figure 2A). In stark contrast, the IN-immunized FLA-ST group demonstrated exceptional protection, with mice recovering to approximately 92.1% of their prechallenge weight as early as 2 dpi, underscoring its potent protective effect.

Paradoxically, the CQ-adjuvanted group, despite eliciting the highest systemic anti-body titers, experienced the most severe and rapid weight loss, with the slowest recovery kinetics among all immunized groups (Figure 2A). More alarmingly, this was the only group to exhibit mortality, with the survival rate decreasing to 80% by 4 dpi (n = 5) (Figure 2B), whereas all the other groups maintained 100% survival throughout the observation period.

**Figure 3:**
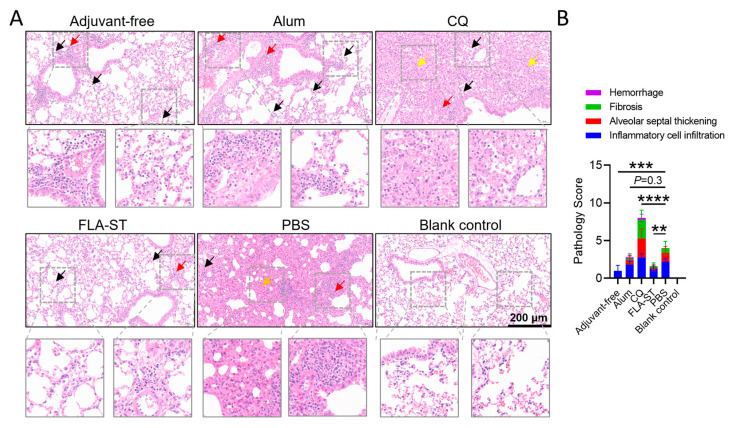
Histopathological assessment of infected lung tissues: (**A**) Representative photomicrographs of H&E-stained lung sections from each group (magnification, 200×); bar = 200 μm. The black arrows indicate the aggregation of inflammatory cells, including granulocytes, eosinophils, and lymphocytes. Red arrows point to focal lymphocytic infiltration. Orange arrows show granulocytic infiltration, while yellow arrows highlight areas of connective tissue hyperplasia. To better visualize the inflammatory infiltrates, selected areas (indicated by boxes in the main images) were digitally magnified to twice the original magnification. (**B**) Double-blind histopathological scores (HPS) of lungs from each group after MP challenge. The data are presented as the mean ± SD and were compared by ANOVA; the mean of each column was compared with the mean of the PBS column. **, *p* < 0.01; ***, *p* < 0.001; ****, *p* < 0.0001. MP, *Mycoplasma pneumoniae*.

To determine whether these clinical outcomes correlated with pathogen control, we quantified the MP burden in the lungs at 7 dpi using CFU assays. As a prerequisite, we confirmed the success of our infection model, as the pulmonary bacterial load was significantly greater in the PBS group than in the blank control group. Compared with the PBS group, both the IM adjuvant-free group and the Alum group exhibited only a modest reduction in bacterial load. In contrast, the pulmonary bacterial burden in the CQ group effectively decreased to approximately 6.5 × 10^5^ CFU/mL in lung tissue. Notably, the FLA-ST group demonstrated the most effective pathogen clearance, reducing the bacterial load to approximately 4.7 × 10^5^ CFU/mL, the lowest level observed across all immunized groups (Figure 2C). Compared with the PBS group, the FLA-ST group exhibited a highly significant decrease in bacterial burden, with an approximately 4-fold decrease in CFU counts (*p* < 0.001). Similarly, compared with the PBS group, the CQ-adjuvanted group also showed a significant decrease in pathogen load. Notably, no statistically significant difference in the bacterial load was observed between the FLA-ST and CQ groups (Figure 2C). Despite similar bacterial clearance, severe lung pathology was observed in the CQ group. Furthermore, one mortality occurred within this group during the post-challenge period (Figure 2B). In contrast, animals in the FLA-ST group did not exhibit such severe outcomes. Furthermore, immunization with the adjuvant-free vaccine or alum-adjuvanted group resulted in a moderate decrease in the pulmonary pathogen load compared with that in the PBS group, although this reduction was less pronounced than that observed in the FLA-ST and CQ groups (Figure 3A). Although no significant difference was observed in the MP load in the BALF as determined by RT-qPCR (Figure 2D), this is likely attributable to the inherent limitations of the BALF sampling method, which primarily lavages the luminal surface and may inefficiently recover pathogens tightly adhering to the epithelium.

Taken together, these data reveal that while high levels of systemic antibodies (in the CQ group) can contribute to bacterial clearance, they are not sufficient for protection and are associated with severe immunopathology. Conversely, mucosal immunization with FLA-ST provides superior, well-rounded protection, characterized by minimal clinical signs and the most efficient clearance of the pathogen from the lungs.

### 3.3. Compared with CQ, FLA-ST Significantly Reduced the Severity of Pulmonary Pathology

A central finding of this study is that the mucosal adjuvant FLA-ST has a superior and comprehensive protective effect against MP infection. Following challenge with 5 × 107 CFU/individual MP, mice in the FLA-ST group exhibited a markedly healthy pulmonary status. Histopathological analysis by H&E staining revealed that the lung architecture in the FLA-ST group remained largely intact, with only minimal and well-controlled inflammatory infiltration and slight alveolar wall thickening observed (Figure 3A). Consistent with this, vaccination with FLA-ST conferred protection against lung immunopathology, as evidenced by a marked reduction in histopathological score (HPS) compared with that in the challenge-only control group (*p* < 0.01; Figure 3B). Although the adjuvant-free group numerically displayed the lowest mean score, there were no statistically significant differences in HPS among the FLA-ST, alum, and adjuvant-free groups. These results directly demonstrate the excellent safety profile of the FLA-ST mucosal immunization strategy. Notably, the CQ group elicited the highest antibody levels overall (Figure 1B–D). However, this potent systemic humoral response did not translate into effective protection and was instead strongly correlated with more severe lung damage. Following pathogen challenge, the CQ group, which generated the highest antibody titers, also displayed the most severe pulmonary immunopathological damage. H&E-stained sections from this group revealed extensive pulmonary congestion and massive inflammatory cell infiltration (Figure 3A). Crucially, these infiltrates were composed predominantly of neutrophils, with a notable secondary population of eosinophils, resulting in a significantly greater HPS than in all the other groups (Figure 3B). Moreover, the observation of significant fibrosis, evidenced by extensive connective tissue proliferation, in the CpG-adjuvanted group is particularly alarming. Fibrosis represents a pathological and often irreversible tissue repair process that leads to organ scarring and long-term loss of function. These findings suggest that while the CpG formulation may offer some level of control over bacterial burden, it does so at the cost of inducing harmful, nonresolving inflammation that promotes tissue remodeling. This risk of inducing permanent lung damage strongly argues against the use of this specific intramuscular CpG formulation for an MP vaccine.

However, the modest efficacy of the CQ adjuvant was offset by the induction of excessive immunopathology. In contrast, compared with the infection control group, the alum adjuvant group showed a mild reduction in immunopathology (Figure 3), although mild to moderate inflammatory infiltration was still present, indicating that the protection conferred was only partial.

### 3.4. Mucosal Immunization with the Inactivated MP Vaccine Combined with FLA-ST Induced Robust IgA Responses

**Figure 4:**
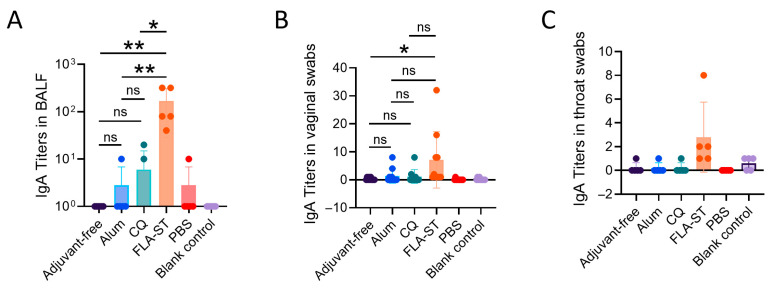
Specific IgA levels in local mucosal sites of mice measured using ELISA after challenge: (**A**) IgA titers in the BALF of mice. (**B**) IgA titers in the vaginal swabs of the mice. (**C**) IgA titers in the throat swabs of the mice. The data are presented as the mean ± SD. Significant differences between the four vaccinated groups were assessed by ANOVA, and the mean of each column was compared with the mean of every other column. ns, not significant; *, *p* < 0.05; **, *p* < 0.01. BALF, bronchoalveolar lavage fluid.

We investigated the ability of vaccines to induce mucosal immunity. Our findings indicate that the success of FLA-ST stems from its unique ability to activate a robust mucosal immune response, establishing a formidable IgA antibody barrier at the primary portal of infection—the respiratory tract. Strikingly, an analysis of the BALF revealed that the FLA-ST group was the only group in which high titers of MP-specific IgA were generated (Figure 4A). This frontline defense, positioned directly at the site of pathogen invasion, is poised to neutralize and clear the pathogen efficiently, thereby obviating the need for a subsequent and potentially damaging systemic inflammatory response. In stark contrast, specific IgA was completely undetectable in the BALF of all the IM-immunized groups, a finding that provides a clear mechanistic explanation for their failure to prevent severe lung immunopathology. Furthermore, the immunogenic reach of FLA-ST extended beyond the respiratory tract, stimulating a widespread mucosal immune network. Significant levels of specific IgA were also detected in distal mucosal sites, including in vaginal and throat swabs (Figure 4B,C). These results demonstrate the potent and systemic nature of the mucosal activation induced by FLA-ST. In conclusion, the advantages of the FLA-ST strategy are multifaceted and mechanistically distinct.

## 4. Discussion

The development of a safe and effective vaccine against MP has remained an elusive goal for decades, with historical efforts being consistently hampered by poor efficacy and the alarming risk of VAERD. The continued absence of a licensed vaccine underscores the magnitude of this challenge. In this study, we directly confront this long-standing impasse. We propose a central thesis: the repeated failures of past inactivated MP vaccines are not an indication of the intrinsic properties of the antigen but rather a predictable consequence of suboptimal adjuvant selection and inappropriate routes of administration. In support of this, we provide compelling evidence that an inactivated whole-cell MP immunogen, when strategically formulated with a rationally chosen mucosal adjuvant and delivered intranasally, can elicit robust, nonpathological, and highly protective immunity. This finding fundamentally challenges the prevailing dogma and offers a new path forward.

A primary finding of our study is the exceptional protective efficacy conferred by the IN-delivered FLA-ST-adjuvanted vaccine. This conclusion is supported by a consistent triad of evidence. Clinically, mice in the FLA-ST group exhibited minimal morbidity, with the fastest weight recovery and lowest overall weight loss postchallenge. Pathologically, this group displayed the most significantly attenuated pulmonary inflammation upon histological examination. Most importantly, from a microbiological standpoint, IN injection with FLA-ST resulted in the most efficient clearance of MP from the lungs. This comprehensive protection profile strongly suggests that generating immunity directly at the site of infection—the respiratory mucosa—is critical for effective defense. FLA-ST, a TLR5 agonist derived from bacterial flagellin, is an excellent mucosal adjuvant [35]. Compared with the CQ group, the IN group immunized with FLA-ST, a Th1-polarizing adjuvant similar to CQ (Figure 1E), was more likely to drive a functional cellular response capable of clearing the pathogen without triggering the excessive inflammation that underlies MP-associated immunopathology. Its principal advantage lies in the widespread expression of TLR5 on the surface of mucosal epithelial cells, including those in the respiratory tract [36]. Consequently, upon IN administration, FLA-ST can directly activate the immune system at the portal of pathogen entry, efficiently inducing a local mucosal immune response spearheaded by secretory IgA (sIgA) while simultaneously stimulating a systemic response (Figure 4) [37]. As the first line of defense against mucosal pathogens, sIgA effectively neutralizes and clears pathogens, fundamentally preventing their adhesion and colonization in the respiratory tract [38]. Therefore, we conclude that the synergistic application of the FLA-ST adjuvant and the mucosal immunization route is the fundamental reason for its superior protective effect, suggesting a highly promising direction for MP vaccine development.

This study also revealed critical and paradoxical findings. Specifically, the CQ composite adjuvant, which has demonstrated excellent performance in inducing Th1-oriented humoral and cellular immunity in response to other vaccines [29,39], induced severe pulmonary immunopathology and even mortality in mice when combined with an inactivated MP antigen. The trend of the Th1/Th2 immune response does not seem to be correlated with the risk of VAERD in response to inactivated MP vaccines. The mucosal adjuvant FLA-ST also induced a Th1-biased immune response similar to that induced by CQ (Figure 1E), which supports our conclusion. Conversely, our findings reveal a significant liability associated with this adjuvant–antigen combination, demonstrating that its potent immunostimulatory capacity can paradoxically precipitate immunopathology when paired with certain immunogens. We posit that this adverse outcome is not attributable to any intrinsic toxicity of the adjuvant itself but rather stems from its exacerbation of the inherent risk of VAERD associated with the inactivated MP antigen. This hypothesis is corroborated by both historical precedent in the literature and the distinct, proinflammatory milieu observed in our study [18,19]. Consistent with this model, the traditional alum adjuvant, which is known to promote a Th2-biased response, failed to confer robust protection and was likewise unable to prevent disease pathology postchallenge. In conclusion, our findings have significant implications for the development of MP vaccines. First, the traditional reliance on serum antibody titers as the primary endpoint may be insufficient to fully assess vaccine efficacy against MP; future studies should place greater emphasis on evaluating mucosal immunity. Second, given the superior performance of FLA-ST and the intranasal route, mucosal adjuvants and delivery methods represent critical future directions for the development of vaccines against MP and other respiratory pathogens. With respect to MP vaccine design, inducing a local immune response directly at the site of infection may offer more effective protection than generating a solely systemic response. Finally, the safety concerns raised by the CQ combination serve as a critical reminder that the use of such potent adjuvants requires careful consideration. Although they can enhance immunogenicity, their effects must be thoroughly evaluated in the context of specific pathogens to avoid exacerbating immunopathology through excessive immune activation. As a limitation of our study’s single-endpoint design, we cannot definitively conclude whether this represents an active induction of immunopathology by the CQ or simply altered kinetics of inflammation resolution compared with the control. For instance, inflammation may have resolved more slowly in this group. Future studies incorporating a time-course analysis are needed to fully dissect the dynamics of the immune response and distinguish between these possibilities. Nevertheless, the combination of high pathological scores and lower body weights strongly suggests a suboptimal safety and efficacy profile for intramuscular CQ in our model. Although this study provides valuable insights, several limitations should be acknowledged. First, a limitation of the current study is the use of Imject™ alum as an adjuvant. This now-discontinued product is a formulation of aluminum hydroxycarbonate and magnesium hydroxide, which has been reported to possess lower immunostimulatory potency than standard aluminum-based adjuvants like Alhydrogel^®^ [40]. Consequently, the immune responses observed in our study might not represent the full potential of our antigen. Future studies will employ a well-characterized, standard adjuvant such as Alhydrogel^®^ to facilitate direct comparisons with other vaccine candidates and to conduct a more comprehensive evaluation of efficacy. Second, the murine model, while widely used for MP research, may not fully recapitulate the immunological features of human infection. Finally, this study did not delve into the detailed mechanistic interactions between the vaccine and adjuvants, nor did it assess long-term protective immunity. Future research will focus on further optimization of the FLA-ST adjuvant formulation and structure, as well as on dose-response relationship studies. By comparing the immunogenicity, safety, and protective efficacy of an inactivated MP vaccine combined with different adjuvant formulations, this study provides a crucial framework for adjuvant selection in the development of inactivated MP vaccines.

## 5. Conclusions

This study demonstrates that the choice of adjuvant or immunization route is critical for developing a safe and effective MP vaccine. We found that while intramuscular immunization with a CQ-adjuvanted formulation induced a robust immune response that reduced pulmonary bacterial loads, it paradoxically caused severe lung pathology. The conventional alum-adjuvanted vaccine, which was also delivered intramuscularly, offered only mediocre protection, and mild to moderate inflammatory infiltration was noted in the lung tissue.

In sharp contrast, a mucosal vaccine adjuvanted with FLA-ST demonstrated superior efficacy. This combination significantly promoted bacterial clearance while concurrently mitigating vaccine-associated lung inflammation, thus presenting the most desirable safety and efficacy profile. In conclusion, this study highlights that an inactivated MP vaccine adjuvanted by FLA-ST and delivered via mucosal immunization represents a promising forward strategy. This strategy is particularly advantageous because it circumvents the potential risk of VAERD, thereby offering a safer and more effective approach in this work.

## Data Availability

All data used during the study are available from the corresponding author upon request.

## References

[1] Kumar S. (2018). Mycoplasma Pneumoniae: A Significant but Underrated Pathogen in Paediatric Community-Acquired Lower Respiratory Tract Infections. Indian J. Med. Res..

[2] Waites K.B., Talkington D.F. (2004). Mycoplasma Pneumoniae and Its Role as a Human Pathogen. Clin. Microbiol. Rev..

[3] Wang X., Li M., Luo M., Luo Q., Kang L., Xie H., Wang Y., Yu X., Li A., Dong M. (2022). Mycoplasma Pneumoniae Triggers Pneumonia Epidemic in Autumn and Winter in Beijing: A Multicentre, Population-Based Epidemiological Study between 2015 and 2020. Emerg. Microbes Infect..

[4] Diaz M.H., Hersh A.L., Olson J., Shah S.S., Hall M., Edens C. (2025). Mycoplasma Pneumoniae Infections in Hospitalized Children—United States, 2018–2024. MMWR Morb. Mortal. Wkly. Rep..

[5] Kutty P.K., Jain S., Taylor T.H., Bramley A.M., Diaz M.H., Ampofo K., Arnold S.R., Williams D.J., Edwards K.M., McCullers J.A. (2019). Mycoplasma Pneumoniae Among Children Hospitalized with Community-Acquired Pneumonia. Clin. Infect. Dis..

[6] Canavan T.N., Mathes E.F., Frieden I., Shinkai K. (2015). Mycoplasma Pneumoniae-Induced Rash and Mucositis as a Syndrome Distinct from Stevens-Johnson Syndrome and Erythema Multiforme: A Systematic Review. J. Am. Acad. Dermatol..

[7] Liong T., Lee K.L., Poon Y.S., Lam S.Y., Kwok K.M., Ng W.F., Lam T.L., Law K.I. (2015). Extrapulmonary Involvement Associated with Mycoplasma Pneumoniae Infection. Hong Kong Med. J..

[8] Salmon P., Rademaker M. (1993). Erythema Multiforme Associated with an Outbreak of Mycoplasma Pneumoniae Function. N. Z. Med. J..

[9] Guleria R., Nisar N., Chawla T.C., Biswas N.R. (2005). Mycoplasma Pneumoniae and Central Nervous System Complications: A Review. J. Lab. Clin. Med..

[10] Hu J., Ye Y., Chen X., Xiong L., Xie W., Liu P. (2022). Insight into the Pathogenic Mechanism of Mycoplasma Pneumoniae. Curr. Microbiol..

[11] Chaudhry R., Ghosh A., Chandolia A. (2016). Pathogenesis of Mycoplasma Pneumoniae: An Update. Indian J. Med. Microbiol..

[12] Georgakopoulou V.E., Lempesis I.G., Sklapani P., Trakas N., Spandidos D.A. (2024). Exploring the Pathogenetic Mechanisms of Mycoplasmapneumoniae (Review). Exp. Ther. Med..

[13] Ferwerda A., Moll H.A., de Groot R. (2001). Respiratory Tract Infections by Mycoplasma Pneumoniae in Children: A Review of Diagnostic and Therapeutic Measures. Eur. J. Pediatr..

[14] Yin Y., Cao B., Wang H., Wang R., Liu Y., Gao Y., Qu J., Han G., Liu Y. (2013). Survey of macrolide resistance in Mycoplasma pneumoniae in adult patients with community-acquired pneumonia in Beijing, China. Chin. J. Tuberc. Respir. Dis..

[15] Wang G., Wu P., Tang R., Zhang W. (2022). Global Prevalence of Resistance to Macrolides in Mycoplasma Pneumoniae: A Systematic Review and Meta-Analysis. J. Antimicrob. Chemother..

[16] Smith C.B., Friedewald W.T., Chanock R.M. (1967). Inactivated Mycoplasma Pneumoniae Vaccine. Evaluation in Volunteers. JAMA.

[17] Jiang Z., Zhou R., Leung P.H.M., Deng Z., Li S. (2022). An Attenuated Multiple Genetic Mutant of Mycoplasma Pneumoniae Imparts Good Immuno-Protection against M. Pneumoniae Pneumonia in BALB/c Mice. Microb. Pathog..

[18] Tamiya S., Yoshikawa E., Ogura M., Kuroda E., Suzuki K., Yoshioka Y. (2020). Vaccination Using Inactivated Mycoplasma Pneumoniae Induces Detrimental Infiltration of Neutrophils after Subsequent Infection in Mice. Vaccine.

[19] Mara A.B., Gavitt T.D., Tulman E.R., Miller J.M., He W., Reinhardt E.M., Ozyck R.G., Goodridge M.L., Silbart L.K., Szczepanek S.M. (2022). Vaccination with Mycoplasma Pneumoniae Membrane Lipoproteins Induces IL-17A Driven Neutrophilia That Mediates Vaccine-Enhanced Disease. NPJ Vaccines.

[20] Jiang Z., Li S., Zhu C., Zhou R., Leung P.H.M. (2021). Mycoplasma Pneumoniae Infections: Pathogenesis and Vaccine Development. Pathogens.

[21] Hausner M., Schamberger A., Naumann W., Jacobs E., Dumke R. (2013). Development of Protective Anti-Mycoplasma Pneumoniae Antibodies after Immunization of Guinea Pigs with the Combination of a P1-P30 Chimeric Recombinant Protein and Chitosan. Microb. Pathog..

[22] Zhu C., Wang S., Hu S., Yu M., Zeng Y., You X., Xiao J., Wu Y. (2012). Protective Efficacy of a Mycoplasma Pneumoniae P1C DNA Vaccine Fused with the B Subunit of Escherichia Coli Heat-Labile Enterotoxin. Can. J. Microbiol..

[23] Mahmood M., Javaid A., Shahid F., Ashfaq U.A. (2021). Rational Design of Multimeric Based Subunit Vaccine against *Mycoplasma Pneumonia*: Subtractive Proteomics with Immunoinformatics Framework. Infect. Genet. Evol..

[24] Vollmer J., Krieg A.M. (2009). Immunotherapeutic Applications of CpG Oligodeoxynucleotide TLR9 Agonists. Adv. Drug Deliv. Rev..

[25] Bode C., Zhao G., Steinhagen F., Kinjo T., Klinman D.M. (2011). CpG DNA as a Vaccine Adjuvant. Expert. Rev. Vaccines.

[26] Klinman D.M., Klaschik S., Sato T., Tross D. (2009). CpG Oligonucleotides as Adjuvants for Vaccines Targeting Infectious Diseases. Adv. Drug Deliv. Rev..

[27] Zhu D., Tuo W. (2016). QS-21: A Potent Vaccine Adjuvant. Nat. Prod. Chem. Res..

[28] Martin L.B.B., Kikuchi S., Rejzek M., Owen C., Reed J., Orme A., Misra R.C., El-Demerdash A., Hill L., Hodgson H. (2024). Complete Biosynthesis of the Potent Vaccine Adjuvant QS-21. Nat. Chem. Biol..

[29] Luan N., Cao H., Wang Y., Lin K., Liu C. (2022). Ionizable Lipid Nanoparticles Enhanced the Synergistic Adjuvant Effect of CpG ODNs and QS21 in a Varicella Zoster Virus Glycoprotein E Subunit Vaccine. Pharmaceutics.

[30] Nelson S.A., Sant A.J. (2021). Potentiating Lung Mucosal Immunity Through Intranasal Vaccination. Front. Immunol..

[31] Longet S., Paul S. (2023). Pivotal Role of Tissue-Resident Memory Lymphocytes in the Control of Mucosal Infections: Can Mucosal Vaccination Induce Protective Tissue-Resident Memory T and B Cells?. Front. Immunol..

[32] Shim J.-U., Lee S.E., Hwang W., Lee C., Park J.-W., Sohn J.-H., Nam J.H., Kim Y., Rhee J.H., Im S.-H. (2016). Flagellin Suppresses Experimental Asthma by Generating Regulatory Dendritic Cells and T Cells. J. Allergy Clin. Immunol..

[33] Mizel S.B., Bates J.T. (2010). Flagellin as an Adjuvant: Cellular Mechanisms and Potential. J. Immunol..

[34] Wilson R.H., Maruoka S., Whitehead G.S., Foley J.F., Flake G.P., Sever M.L., Zeldin D.C., Kraft M., Garantziotis S., Nakano H. (2012). The Toll-like Receptor 5 Ligand Flagellin Promotes Asthma by Priming Allergic Responses to Indoor Allergens. Nat. Med..

[35] Cui B., Liu X., Fang Y., Zhou P., Zhang Y., Wang Y. (2018). Flagellin as a Vaccine Adjuvant. Expert Rev. Vaccines.

[36] Hayashi F., Smith K.D., Ozinsky A., Hawn T.R., Yi E.C., Goodlett D.R., Eng J.K., Akira S., Underhill D.M., Aderem A. (2001). The Innate Immune Response to Bacterial Flagellin Is Mediated by Toll-like Receptor 5. Nature.

[37] Pino O., Martin M., Michalek S.M. (2005). Cellular Mechanisms of the Adjuvant Activity of the Flagellin Component FljB of *Salmonella enterica* Serovar Typhimurium to Potentiate Mucosal and Systemic Responses. Infect. Immun..

[38] Li Y., Jin L., Chen T. (2020). The Effects of Secretory IgA in the Mucosal Immune System. BioMed Res. Int..

[39] Luan N., Cao H., Wang Y., Lin K., Liu C. (2022). LNP-CpG ODN-Adjuvanted Varicella-Zoster Virus Glycoprotein E Induced Comparable Levels of Immunity with Shingrix^TM^ in VZV-Primed Mice. Virol. Sin..

[40] Cain D.W., Sanders S.E., Cunningham M.M., Kelsoe G. (2013). Disparate Adjuvant Properties among Three Formulations of “Alum”. Vaccine.

